# Cyclin D1 is Associated with Radiosensitivity of Triple-Negative Breast Cancer Cells to Proton Beam Irradiation

**DOI:** 10.3390/ijms20194943

**Published:** 2019-10-07

**Authors:** Changhoon Choi, Sohee Park, Won Kyung Cho, Doo Ho Choi

**Affiliations:** 1 Department of Radiation Oncology, Samsung Medical Center, Seoul 06351, Korea; chchoi93@gmail.com (C.C.); psh3842@nate.com (S.P.); wklove.cho@samsung.com (W.K.C.); 2 Department of Radiation Oncology, Sungkyunkwan University School of Medicine, Seoul 06351, Korea

**Keywords:** breast cancer, proton therapy, relative biological effectiveness, CDK4/6 inhibitor, cyclin D1

## Abstract

Proton therapy offers a distinct physical advantage over conventional X-ray therapy, but its biological advantages remain understudied. In this study, we aimed to identify genetic factors that contribute to proton sensitivity in breast cancer (BC). Therefore, we screened relative biological effectiveness (RBE) of 230 MeV protons, compared to 6 MV X-rays, in ten human BC cell lines, including five triple-negative breast cancer (TNBC) cell lines. Clonogenic survival assays revealed a wide range of proton RBE across the BC cell lines, with one out of ten BC cell lines having an RBE significantly different from the traditional generic RBE of 1.1. An abundance of cyclin D1 was associated with proton RBE. Downregulation of RB1 by siRNA or a CDK4/6 inhibitor increased proton sensitivity but not proton RBE. Instead, the depletion of cyclin D1 increased proton RBE in two TNBC cell lines, including MDA-MB-231 and Hs578T cells. Conversely, overexpression of cyclin D1 decreased the proton RBE in cyclin D1-deficient BT-549 cells. The depletion of cyclin D1 impaired proton-induced RAD51 foci formation in MDA-MB-231 cells. Taken together, this study provides important clues about the cyclin D1-CDK4-RB1 pathway as a potential target for proton beam therapy in TNBC.

## 1. Introduction

Breast cancer (BC) is the most frequently diagnosed cancer in women worldwide, and it continues to be one of the leading causes of cancer-related deaths. The standard treatment for BC combines surgery, radiotherapy, chemotherapy, and hormone therapy, depending on the stage, grade, and molecular subtype of the tumor. Radiation therapy, a recommended treatment for BC, is a local treatment modality that uses high-energy photons or particles to kill cancer cells. Adjuvant radiotherapy following mastectomy or lumpectomy is routinely used because it reduces cancer recurrence [1,2].

Triple-negative breast cancer (TNBC) is a BC subtype that lacks the estrogen receptor (ER), progesterone receptor (PR), and overexpression of human epidermal growth factor receptor 2 (HER2) [3]. TNBCs, which are 15–20% of all BCs, produce highly aggressive and heterogeneous tumors and are associated with a poor prognosis. Currently, platinum-based chemotherapy remains the mainstay for treatment because no targeted therapy is available for TNBC. Whether the benefit of radiotherapy varies by molecular subtype remains unknown [4]. Mutations in *BRCA1*, a tumor suppressor gene that is involved in DNA damage repair, such as homologous recombination (HR), are prevalent in TNBC patients. In addition, “BRCA-ness,” which refers to the biological characteristics of altered BRCA functionality without *BRCA* mutations, is also predominantly detected in TNBC, compared to other BC types [5].

As a highly conformal approach, proton beam therapy could be a better option than conventional photon radiotherapy. The physical properties of proton beams, such as the Bragg peak, have been well established, and their biological properties have been regarded as similar to those of photons [6]. Proton beam therapy simply uses a fixed relative biological effectiveness (RBE) value of 1.1 relative to megavoltage X-rays and has been proven to be effective in numerous in vivo and in vitro studies [6,7]. However, recent radiobiological data from lung cancer cells [8], head and neck cancer cells [9], and liver cancer cells [10] have shown that proton beams exert profoundly different effects on cancer cells from photons, depending on the genetic background. Alterations to DNA damage-repair genes and Fanconi anemia genes affect proton sensitivity [8,10,11,12]. Some targeted agents also modulate the effects of proton beam therapy. For example, a PARP inhibitor changed proton radiosensitivity in lung and pancreatic cancers [13] and a histone deacetylase inhibitor increased the sensitization of hepatocellular carcinoma cells to protons [14].

In BC treatment, compared to conventional radiotherapy, proton therapy may have the higher technical advantage of reducing the risk of radiation-associated damage to the heart and lungs [15,16]. However, the biological advantage of protons over photons in BC treatment has not been determined. Comprehensive profiling of BC molecular signatures helps guide clinical decisions regarding treatment strategies [17]. However, the genetic factors or signaling pathways that may affect the sensitivity of BC to proton therapy remain unknown. To address these questions, we determined the RBE of 230 MeV proton therapy compared to 6 MV X-ray treatment in human BC cell lines and dissected the signaling pathway complexity in response to proton irradiation.

## 2. Results

### 2.1. Screening of Human BC Cell Lines to X-ray and Proton Radiosensitivity

**Figure 1:**
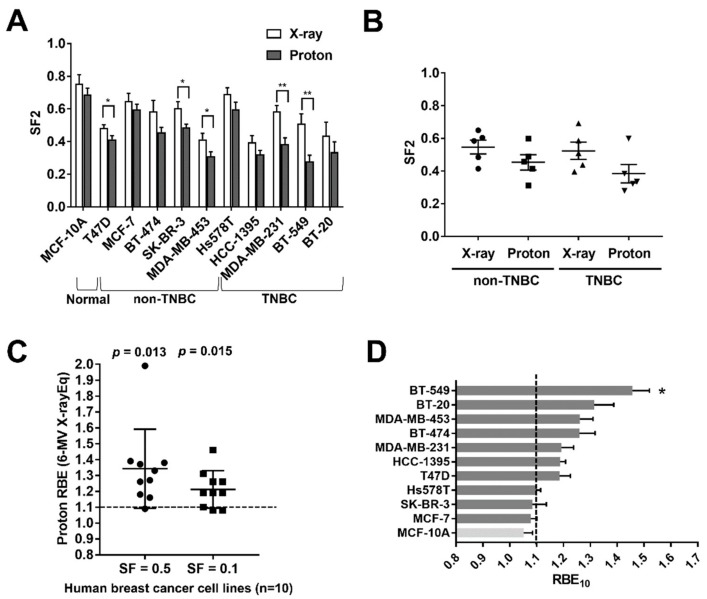
Comparison of radiosensitivity and proton relative biological effectiveness (RBE) in breast cancer cell lines. (**A**) Comparison of the survival fraction at 2 Gy (SF2) of X-ray or proton treatment across 11 human breast cancer cell lines. Data are presented as the mean ± SEM from three independent experiments and the differences between proton and X-ray results are evaluated by a student *t*-test; * *p* < 0.05; ** *p* < 0.01. (**B**) Comparison of SF2 for X-rays and protons between non-TNBC and TNBC groups. No significant differences between groups are seen. (**C**) Proton RBE values relative to megavoltage X-rays for ten BC cell lines at SF = 0.5 and SF = 0.1. (**D**) Comparison of RBE_10_ values for BC cell lines. RBE_10_ was calculated as the ratio of megavoltage X-ray doses to proton doses that yield 10% survival. RBE of each cell line was compared to 1.10 using a one-sample *t*-test; * *p* < 0.05.

A recent study showed that human BC cell lines have a diverse range of radiation sensitivity to photons, such as X-rays, as determined by the survival fraction (SF) obtained from a clonogenic assay at 2 Gy (SF2) [18]. To compare the RBE of protons over photons, we irradiated 11 human breast cell lines with 6 MV X-rays and 230 MeV protons, which are therapeutic radiations that are being used clinically, and determined the radiation dose-response curves (Figure S1). The human breast cell lines tested in this study included a normal, immortalized breast epithelial cell line, five TNBC cell lines, and five non-TNBC cell lines. The clonogenic assay revealed that the human breast cell lines had diverse sensitivity to either protons or X-rays (Figure 1A). The mean SF2 values ranged from 0.39 to 0.75 for X-rays and from 0.28 to 0.68 for protons. There were no statistically significant differences in radiosensitivity between the non-TNBC groups and the TNBC groups, although the mean SF2 values for protons were lower than those for X-rays (Figure 1B).

In non-small-cell lung cancer (NSCLC), head and neck cancer, and liver cancer, the RBE of protons over X-rays is diverse owing to genetic heterogeneity [8,9,10]. Using BC cell lines, we determined the proton RBE_50_ and RBE_10_ defined as the ratio of photon to proton doses yielding the same biological endpoint, a SF of 0.5 and 0.1, respectively. We used clinical 6 MV X-rays as a reference for photon radiation because this megavoltage X-rays are currently being used to treat patients and are proven to be equivalent to ^137^Cs [19]. The mean RBE values of protons relative to 6 MV X-rays were 1.34 and 1.21 at SF = 0.5 and 0.1, respectively, which were significantly different from the traditional generic proton RBE of 1.1 (Figure 1C). Based on a SF of 0.1, seven out of eleven cell lines had a higher RBE_10_ than 1.1, and BT-549 cells were the only ones whose RBE_10_ was significantly different from 1.1 (*p* < 0.05, one-sample *t*-test; Figure 1D). Thus, our data indicate that proton RBE for BC cell lines covered a wide range, similar to the effects in other cancer cell lines, possibly as a result of diversity in genetic backgrounds.

### 2.2. Involvement of the Cyclin D1/CDK4/RB Pathway in Proton Radiosensitivity

**Figure 2:**
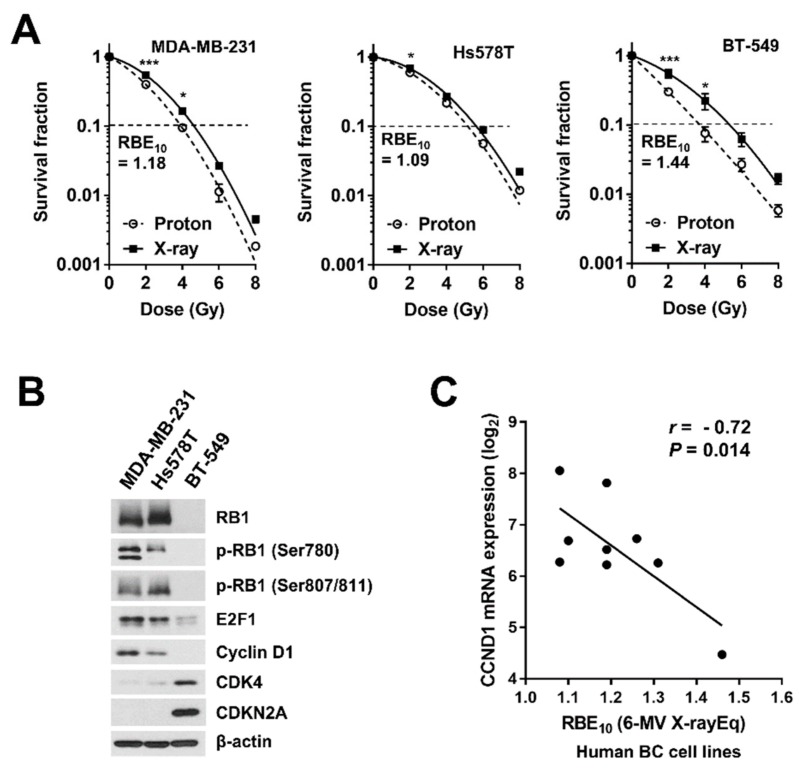
The possible implication of the RB1 pathway in the proton sensitivity of TNBC cells. (**A**) Dose-response curves of three TNBC cell lines, MDA-MB-231, Hs578T, and BT-549, which were irradiated with X-rays or protons. Data are presented as the mean ± SEM for three independent experiments. Curves were fitted with a linear quadratic model. The differences are evaluated by a two-way ANOVA; * *p* < 0.05; *** *p* < 0.001. Dashed lines indicate SF = 0.1. (**B**) Expression of RB pathway-related proteins among the three TNBC cell lines. Western blot analysis was performed as described in the materials and methods. β-Actin was used as a loading control. (**C**) Scatter plot for correlation between RBE_10_ values and *CCND1* mRNA expression in human BC cell lines. Log2-transformed RMA values were obtained from the Cancer Cell Line Encyclopedia (CCLE) database. Pearson’s correlation coefficients (*r*) between gene expression and proton RBE_10_ across all samples were presented.

To identify the genetic factors that may influence proton radiosensitivity in human BC, we searched for genetic mutations previously reported in BT-549 cells, which had the highest RBE_10_ value (Figure 1D). Several studies have shown that BT-549 cells have a loss of RB1, a key regulator of G1/S cell cycle progression [20,21,22]. We investigated the cyclin D1/CDK4/RB1 pathway in three TNBC cell lines, MDA-MB-231, Hs578T, and BT-549 cells, which had different RBE_10_ values (Figure 2A), and found that BT-549 cells had a pattern of expression for RB1 pathway proteins distinct from those of Hs578T and MDA-MB-231 cells, with the loss of RB1 and cyclin D1 and high expression of CDK4 and CDKN2A (Figure 2B). To determine whether there was a correlation between mRNA expression of these RB1 pathway genes and proton RBE, we downloaded gene expression profile data for the BC cell lines from the Cancer Cell Line Encyclopedia (CCLE) portal (2 January 2019, http://portals.broadinstitute.org/ccle). A correlation analysis revealed that expression of only the *CCND1* gene encoding cyclin D1 was negatively correlated with the proton RBE_10_ in ten BC cell lines (Pearson’s correlation coefficient, *r* = −0.72, *p* = 0.014; Figure 2C). Expression of *CDK4* mRNA showed a positive correlation with proton RBE_10_ (*r* = 0.52, *p* = 0.061; Figure S2A). To confirm whether the negative correlation was observed in other cancer types, we obtained proton RBE values (relative to ^60^Co gamma-ray) of 17 human NSCLC cell lines from a previously published study [8] and found a similar negative correlation between *CCND1* mRNA and proton RBE (*r* = −0.55, *p* = 0.010; Figure S2B). These data suggest that the cyclin D1/CDK4/RB1 pathway may be implicated in proton RBE in BC cells.

### 2.3. Effects of RB1 Depletion on Proton-Induced Apoptosis and Clonogenic Survival

**Figure 3:**
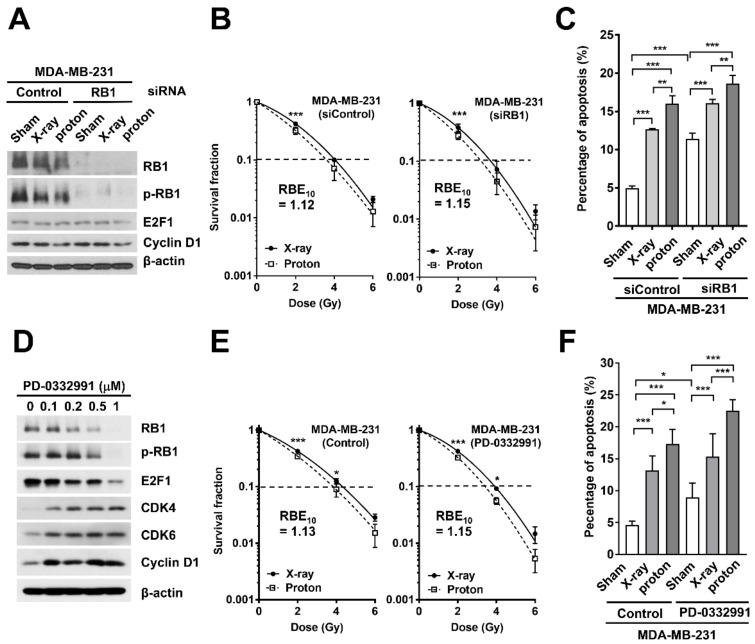
RB1 downregulation via siRNA or CDK4/6 inhibitor does not increase proton RBE in MDA-MB-231 cells. (**A**) Western blot analysis confirms siRNA-mediated RB1 knockdown in MDA-MB-231 cells. β-Actin was used as a loading control. Neither radiation treatment nor RB1 knockdown affected the expression of cyclin D1 (upstream effector) or E2F1 (downstream of RB1). (**B**) Effect of RB1 depletion on the clonogenic survival of MDA-MB-231 cells after X-ray or proton irradiation. Data are presented as the mean ± SEM of two independent experiments and the differences are evaluated by a two-way ANOVA; *** *p* < 0.001. (**C**) The RB1 depletion augmented radiation-induced apoptosis. Apoptotic cell death was assessed by flow cytometry as described in the materials and methods. Data are presented as the mean ± SEM of two independent experiments. The differences were evaluated by a two-way ANOVA; ** *p* < 0.01; *** *p* < 0.001. (**D**) PD-0332991, a selective CDK4/6 inhibitor, decreased total RB and its phosphorylated form in a dose-dependent manner. β-Actin was used as a loading control. (**E**) The effect of PD-0332991 on clonogenic survival. MDA-MB-231 cells were pre-treated with 100 nM PD-0332991 for 3 h, followed by irradiation with the indicated doses of X-rays or protons. Data are presented as the mean ± SEM of two independent experiments and the differences are evaluated by a two-way ANOVA; * *p* < 0.05; *** *p* < 0.001. (**F**) PD-0332991 enhanced proton radiation-induced apoptosis. MDA-MB-231 cells were pre-treated with 500 nM PD-0332991, followed by exposure to 4 Gy of X-rays or protons. Data are presented as the mean ± SEM of three independent experiments and the differences are evaluated by a two-way ANOVA; * *p* < 0.05; ** *p* < 0.01; *** *p* < 0.001.

To test the effects of the loss of RB1 on proton RBE, we depleted RB1 in RB1-proficient MDA-MB-231 cells using siRNA transfection and determined the radiosensitivity. Western blot analysis confirmed siRNA-mediated depletion of RB1 (Figure 3A), which did not affect the expression of E2F1 and cyclin D1. The clonogenic survival assay showed that the RB1 siRNA decreased clonogenic survival after both proton and X-ray irradiation when compared to the control siRNA (Figure 3B). However, the RB1 depletion did not increase the RBE_10_. Apoptosis analysis using flow cytometry showed that proton irradiation increased the apoptotic cell population, from 4.97% to 16.0%, to a greater extent than that by X-ray irradiation (12.7%, two-way ANOVA, *p* < 0.01; Figure 3C). The RB1 siRNA also induced apoptosis (11.4%), but its combination with either X-rays or protons did not cause any additive effects on apoptosis (Figure 3C).

Next, we determined whether CDK4/6-mediated RB1 phosphorylation, another mechanism for RB1 regulation, is related to proton RBE. For this, we utilized a selective CDK4/6 inhibitor, PD-0332991 (also known as palbociclib). As expected, PD-0332991 decreased the expression of phosphorylated RB1 and total RB1 in MDA-MB-231 cells in a concentration-dependent manner (Figure 3D). It also decreased the expression of E2F1, while it increased the expression of CDK4, CDK6, and cyclin D1. When combined with radiation treatments, the PD-0332991 lowered clonogenic survival when compared to that in the untreated cells (Figure 3E). However, it did not increase the RBE_10_. PD-0332991 induced apoptosis in MDA-MB-231 cells (*p* < 0.001; Figure 3F), but again, its combination with either X-rays or protons did not show any additive effects (Figure 3F). These data indicate that RB1 depletion using either siRNA or CDK4/6 inhibitor increased radiosensitivity but not proton RBE.

### 2.4. Effects of Cyclin D1 Depletion on Proton-Induced Apoptosis and Clonogenic Survival

**Figure 4:**
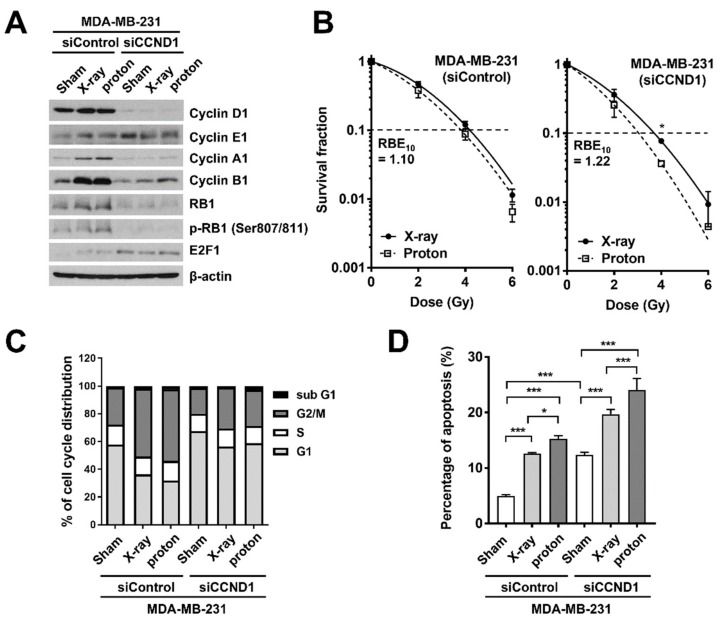
Cyclin D1 depletion increases proton RBE in MDA-MB-231 cells. (**A**) Western blot analysis confirms siRNA-mediated cyclin D1 knockdown in MDA-MB-231 cells. β-Actin was used as a loading control. (**B**) Effect of cyclin D1 knockdown on the clonogenic survival of MDA-MB-231 cells after X-ray or proton irradiation. The cells were transfected with either control siRNA or *CCND1* siRNA, followed by irradiation with the indicated doses of X-rays or protons. Data are presented as the mean ± SEM of two independent experiments and the differences are evaluated by a two-way ANOVA; * *p* < 0.05. (**C**) Effect of cyclin D1 knockdown on cell cycle progression in MDA-MB-231 cells. (**D**) Cyclin D1 knockdown promoted radiation-induced apoptosis. MDA-MB-231 cells transfected with control siRNA or cyclin D1 siRNA were irradiated with 4 Gy of X-rays or protons. Apoptosis was determined using flow cytometry 72 h post-irradiation. Data are presented as the mean ± SEM of two independent experiments and the differences are evaluated by a two-way ANOVA; * *p* < 0.05; *** *p* < 0.001.

Based on the fact that cyclin D1 mRNA levels were negatively correlated with proton RBE (Figure 2C), we determined the effects of cyclin D1 depletion on proton RBE in MDA-MB-231 cells. Western blots confirmed the siRNA-mediated depletion of cyclin D1 in the MDA-MB-231 cells (Figure 4A). Depletion of cyclin D1 decreased expression of cyclin A1, cyclin B1, and RB1, while increasing the expression of E2F1 and cyclin E1 (Figure 4A). A clonogenic survival assay revealed that cyclin D1 depletion increased not only proton sensitivity but also proton RBE_10_ (from 1.10 to 1.22; Figure 4B). Radiation treatment induced G2/M arrest and cyclin D1 depletion increased cell populations at the G1 phase (Figure 4C). Cyclin D1 siRNA increased apoptotic cell death from 4.58% to 12.7% (*p* < 0.001), and it had an additive effect with radiation, with the combined proton treatment being more effective than that with X-rays (23.3% versus 20.2%, a two-way ANOVA, *p* < 0.001; Figure 4D).

**Figure 5:**
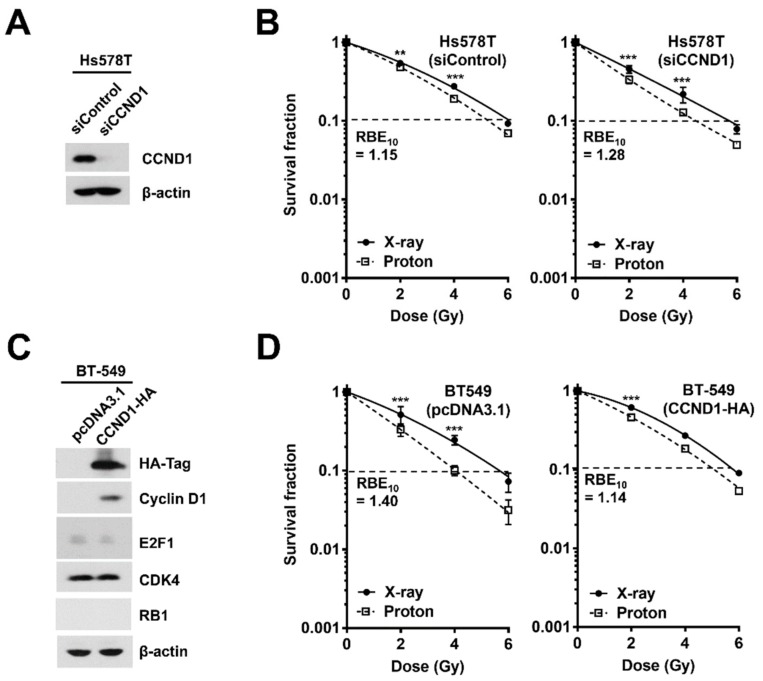
Depletion or re-expression of cyclin D1 affects proton RBE in TNBC cells. (**A**) The knockdown of cyclin D1 via siRNA in Hs578T cells. (**B**) Depletion of cyclin D1 increased proton RBE in the Hs578T cell line, as judged by a clonogenic survival assay. Data are presented as the mean ± SEM of two independent experiments and the differences are evaluated by a two-way ANOVA; ** *p* < 0.01; *** *p* < 0.001. Dashed lines indicate SF = 0.1. (**C**) Western blot analysis confirmed transient overexpression of HA-tagged cyclin D1 in BT-549 cells. Ectopic expression of cyclin D1 did not affect expression of other genes, such as *RB1* and *E2F1*. (**D**) Overexpression of cyclin D1 in BT-549 cells decreased RBE_10_ from 1.40 to 1.14. Data are presented as the mean ± SEM of two independent experiments and the differences are evaluated by a two-way ANOVA; *** *p* < 0.001.

Next, to confirm whether the role of cyclin D1 in proton RBE is also observed in other TNBC cells, we depleted cyclin D1 in Hs578T cells, which seemed more radioresistant than MDA-MB-231 cells (Figure 5A). In Hs578T cells, cyclin D1 depletion also increased proton RBE_10_ from 1.15 to 1.28 (Figure 5B). To further support the role of cyclin D1 in proton RBE in TNBC cells, we overexpressed HA-tagged cyclin D1 in BT-549 cells, in which cyclin D1 expression was almost absent (Figure 5C). Ectopic expression of cyclin D1 did not affect the protein expression of other RB1-related genes, including *E2F1*, *CDK4*, and *RB1* (Figure 5C). Transfection with a pcDNA3.1 empty vector in BT-549 cells did not alter radiosensitivity or proton RBE; RBE_10_ of pcDNA3.1-transfected BT-549 cells was 1.40, which was very close to that of the untreated BT-549 cells (Figure 5D). In contrast, overexpression of cyclin D1 in BT-549 cells decreased not only radiosensitivity but also proton RBE_10_ from 1.40 to 1.14 (Figure 5D). These data suggest that cyclin D1 may be a critical factor for determining proton RBE in TNBC cells.

### 2.5. Effects of Cyclin D1 Depletion on Proton-Induced RAD51 Foci Formation

**Figure 6:**
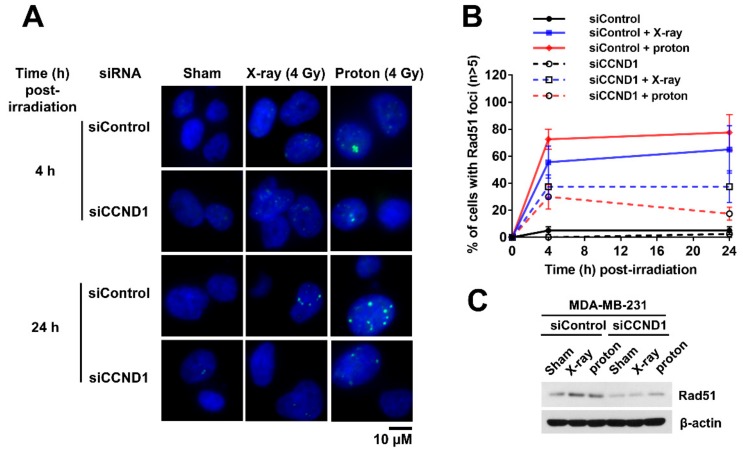
Depletion of cyclin D1 impairs radiation-induced formation of RAD51 foci. (**A**) Representative images of radiation-induced RAD51 foci in MDA-MB-231 cells. The MDA-MB-231 cells were transfected with either control siRNA or *CCND1* siRNA, followed by irradiation with 4 Gy of X-rays or protons. The cells were fixed at the indicated times and stained with DAPI (blue) and RAD51 (green), as described in the materials and methods. (**B**) Kinetics of cells with RAD51 foci (*n* ≥ 5) after 4 Gy of X-rays or protons. Data are presented as the mean ± SEM of two independent experiments (*n* = 40). (**C**) Depletion of cyclin D1 decreased RAD51 expression. β-Actin was used as a loading control.

To gain insight into how the depletion of cyclin D1 increased proton RBE, we determined the kinetics of the RAD51 foci formation after X-ray or proton irradiation in MDA-MB-231 cells. RAD51 is a surrogate marker for proton sensitivity [11,12]. Immunofluorescence images showed that RAD51 foci were barely observed in unirradiated samples, but their numbers increased with 4 Gy of radiation (Figure 6A). Proton irradiation induced RAD51 foci formation more effectively than that by X-rays, although the difference was not statistically significant (Figure 6B). Depletion of cyclin D1 via *CCND1* siRNA decreased RAD51 foci numbers in both proton- and X-ray-irradiated cells (Figure 6A,B). The depletion of cyclin D1 also decreased RAD51 expression (Figure 6C). Thus, these data suggest that depletion of cyclin D1 diminished the abundance of RAD51, attenuating its foci formation in response to proton irradiation.

## 3. Discussion

Currently, biomarker-driven precision medicine is prevalent in medical oncology. BC, with its well-defined predictive biomarkers, is a good example of the application of precision medicine [3,17]. In TNBC management, systemic chemotherapy is the mainstay, and the benefits of radiation therapy remain unclear. *BRCA1/2* gene mutations or BRCA-ness are molecular characteristics of the TNBC subtype, leading to HR deficiency. Based on previous preclinical studies that protons may be more effective than photons for HR-deficient cancers, we aimed to investigate the biological effect of proton therapy on TNBC management.

For patients with BC, compared to conventional X-ray radiotherapy, proton beam therapy is now receiving more attention because it may theoretically lower cardiovascular toxicity [15,16]. Despite the dosimetric advantage of proton therapy over conventional photon therapy, its biological advantages have not yet been addressed in BC. As in other types of cancer, a generic RBE value of 1.1 is used for BC treatment, but recent radiobiology studies argue that the RBE value is oversimplified and have proposed incorporating genetic factors into the RBE [6,8,23]. A previous study using NSCLC cell lines showed that five out of seventeen NSCLC cell lines (29.4%) had RBE values that were significantly different from 1.1 [8,24]. Our current RBE screening in BC cell lines revealed that seven out of ten cell lines had proton RBE_10_ values larger than 1.1 (Figure 1C). However, only BT-549 cells had significantly different RBE values.

The RB1 pathway, a key player in cell cycle progression, is frequently deregulated in many cancers, including BC, and it is considered to be a therapeutic target [25,26,27]. The canonical RB1 pathway consists of RB1, cyclin D1, CDK4/6, p16, and the E2F family. RB1 binds directly to E2F family proteins and suppresses their transcriptional activity. The accumulation of cyclin D1 in response to mitotic cues and subsequent activation of CDK4/6 inactivates RB1 through hyper-phosphorylation of multiple residues, resulting in cell cycle progression [28]. The deregulation of CDK4/6 by cyclin D1 amplification is often seen in ER-positive BC, whereas the loss of the *RB1* gene is more prevalent in TNBC [26,27]. Mutations in *RB1* and *CDKN2A/p16* are mutually exclusive, where the loss of CDKN2A/p16 retains wild-type RB1, and the loss of RB1 results in high expression of CDKN2A/p16. In our study, the former scenario was present in MDA-MB-231 and Hs578T cells and the latter was observed in BT-549 cells (Figure 2B). Correlation analyses between expression profiles of RB1 pathway genes and radiosensitivity in BC cell lines revealed a strong correlation between *CCND1* and RBE_10_, with high *CCND1* expression being correlated with low RBE_10_ (Figure 2C). Based on these data, we speculated that the RB1 pathway may be involved in the responsiveness of BC to protons.

Loss of RB1 is associated with good responses to neoadjuvant chemotherapy in BC [26] and RB1-negative TNBC cells are highly sensitive to gamma-irradiation [20]. RB1 depletion conferred increased radiosensitivity in BC and prostate cancer by altering DNA damage repair, thereby inducing apoptosis [29,30]. Our data using TNBC cells showed that the knockdown of RB1 mildly increased the radio-responsiveness of MDA-MB-231 cells to proton therapy via apoptosis induction, but it did not increase proton RBE (Figure 3B). Similarly, the treatment with a CDK4/6 inhibitor increased radiosensitivity of MDA-MB-231 cells but not proton RBE, even though the CDK4/6 inhibitor decreased the abundance of RB1 (Figure 3E). Together, the abundance of RB1 protein may not be a determinant of proton RBE in BC.

Since *CCND1* mRNA was negatively correlated with proton RBE in two types of cancer, BC and NSCLC, we chose cyclin D1 as the next target. The knockdown of cyclin D1 not only increased the proton radiosensitivity but also increased the proton RBE in two different TNBC cell lines (Figure 4A and Figure 5A). Conversely, ectopic expression of cyclin D1 in cyclin D1-deficient BT-549 cells decreased proton RBE_10_ to a value close to that of MDA-MB-231 cells (Figure 5D). The depletion of cyclin D1 decreased phosphorylated RB1 levels (Figure 4A) and thus induced G1 phase arrest (Figure 4C), suggesting that cell cycle arrest may facilitate proton sensitization. However, it may not be a plausible mechanism because cell cycle arrest was also induced by a CDK4/6 inhibitor or direct depletion of RB1. Besides cell cycle regulation, cyclin D1 contributes to DNA repair in human cervical carcinoma, lung cancer [31], prostate cancer [32], and esophageal cancer cells [33]. Depletion of cyclin D1 blocks RAD51 recruitment to double-strand break (DSB) sites, resulting in radiosensitization. Consistently, our data showed that cyclin D1 was necessary for RAD51 foci formation at the proton-induced DNA damage sites in TNBC cells (Figure 6). Since proton-induced DNA damage requires the HR pathway [8,11], the loss of cyclin D1 may likely impair RAD51-mediated effects through the HR pathway, thereby resulting in an increased proton RBE. Nonetheless, the RBE value of cyclin D1-depleted MDA-MB-231 cells remained lower than that of BT-549 cells. This suggests that there may be a compensatory mechanism, such as cyclin E1-induced expression by cyclin D1 depletion (Figure 5A), which may cause CDK2 activation.

In BC treatment, personalized medicine has become a reality, with approved gene expression-based assays such as Oncotype DX and MammaPrint. They are helping physicians predict cancer recurrences and make therapeutic decisions. Genomic diversity and heterogeneity in TNBC increase the necessity for identifying genomic classifiers to predict treatment responses. Although radiation therapy is a modality in BC management, the development of gene signatures to predict the responses to radiation therapy remains limited to preclinical models [18], and these have not been applied to proton therapy. Our data herein suggest that *CCND1,* among RB1 pathway-related genes, could be implicated in proton sensitivity and RBE. These findings will need to be further validated in animal models and the feasibility of *RB1* gene signatures for predicting proton therapy responses will also need further investigation in TNBC patient cohorts. Proton RBE increases at the distal end of SOBP, due to a steep increase in linear energy transfer (LET) [8]. Considering we obtained the RBE data using proton beams at the mid-SOBP, further analysis would be required to determine the effect of LET on cyclin D1-depenent modification in proton RBE.

## 4. Materials and Methods

### 4.1. Cell Culture

Ten human BC cell lines (T47D, MCF-7, BT-474, SK-BR-3, MDA-MB-453, Hs578T, MDA-MB-231, HCC-1395, BT-20, and BT-549) and MCF-10A, a normal epithelial breast cell line, were used for this study. MCF-7, MDA-MB-231, BT-549, BT-20, and MCF-10A were purchased from the American Type Culture Collection (ATCC, Manassas, VA, USA). T47D, BT-474, SK-BR-3, MDA-MB-453, Hs578T, and HCC-1395 were purchased from the Korean Cell Line Bank (Seoul National University, Seoul, Korea). T47D, BT-474, SK-BR-3, HCC-1395, and BT-549 were cultured in Roswell Park Memorial Institute (RPMI) medium supplemented with 10% fetal bovine serum (FBS) and 1× Antibiotic-Antimycotic (AA, Gibco, Carlsbad, CA, USA). MDA-MB-453, MDA-MB-231, and BT-20 cells were cultured in Dulbecco’s modified Eagle’s medium (DMEM) supplemented with 10% FBS and 1 × AA. Hs578T cells were cultured in DMEM supplemented with 10% FBS, 1 × AA, and 25 mM HEPES. MCF-7 was cultured in minimum essential medium (MEM) supplemented with 10% FBS and 1 × AA. MCF-10A cells were cultured in DMEM/F-12 medium supplemented with 5% horse serum, 1 × AA, 20 ng/mL hEGF, 0.5 mg/mL hydrocortisone, 100 ng/mL cholera toxin, and 10 µg/mL insulin. Cultures were maintained at 37 °C in a humidified atmosphere with 5% CO_2_.

### 4.2. Reagents and Antibodies

PD-0332991 (palbociclib), a selective CDK4/6 inhibitor, was purchased from Sigma Aldrich (St. Louis, MO, USA). Anti-phospho-H2AX (Ser139) antibodies were purchased from Millipore (Billerica, MA, USA). RB1, phospho-RB1 (Ser780; Ser807/811), E2F1, cyclin B1, cyclin D1, cyclin E1, CDK4, and CDK6 antibodies were purchased from Cell Signaling Technology (Danvers, MA, USA). Anti-β-actin antibodies were purchased from Sigma Aldrich (St. Louis, MO, USA). Anti-RAD51, cyclin A1, and CDKN2A antibodies were purchased from Santa Cruz Biotechnology (Santa Cruz, CA, USA).

### 4.3. Irradiation Experiments

BC cells were irradiated with clinical radiation beams as previously reported [10,14]. For X-ray irradiation, the cells were placed under a 2 cm thick solid water phantom and exposed to various doses of 6 MV photons. X-rays were delivered using a Varian Clinac 6EX linear accelerator (Varian Medical Systems, Palo Alto, CA, USA) with a dose rate of 3.96 Gy/min. The absolute X-ray dose calibration was done based on a TG-51 protocol with Gafchromic film to a 1% accuracy. For proton irradiation, the cells were positioned in a mid-spread-out Bragg peak (SOBP) of 11.2 cm width and irradiated with 230 MeV proton beams at a dose rate of 2.14 Gy/min. Proton beams were delivered by a proton therapy machine (Sumitomo Heavy Industries, Tokyo, Japan) using the wobbling technique [34]. The absolute proton beam dose was verified to 1% accuracy using the TRS-398 dosimetry protocol. The physical proton dose was used for all experiments, and there was no correction for an RBE of 1.1, as is the clinical practice.

### 4.4. Clonogenic Survival Assays

The radiosensitivity of each human BC cell line was determined using a clonogenic survival assay, as previously described [35]. The exponentially growing cells were plated 18 to 24 h before irradiation, followed by exposure to X-rays or protons with physical doses of 0, 2, 4, 6, and 8 Gy. After 7–14 days of incubation, the cells were fixed in 98% ethanol and stained with 0.5% crystal violet (Sigma-Aldrich). Colonies with at least 50 cells were counted. The SF was calculated by dividing the plating efficiency (ratio of the number of surviving colonies to the number of plated cells) of the irradiated cells with the plating efficiency of unirradiated cells. The survival curves were fitted using a linear-quadratic model (SF = exp(−αD − βD^2^), where SF is the survival fraction and D is the absorbed dose) using GraphPad Prism 7.02 (GraphPad Software, La Jolla, CA, USA) or an in-house RBE analysis program built with MATLAB code [36]. Proton RBE was defined as the ratio of physical 6-MV X-ray dose to proton dose that yielded the same biological outcome.

### 4.5. Transfections

For gene knockdown experiments, control siRNA (sc-37007), and RB1- and cyclin D1-specific siRNAs (sc-29468 and sc-29286, respectively) were purchased from Santa Cruz Biotechnology. For overexpression experiments, a pcDNA-cyclin-D1-HA plasmid (Addgene, Watertown, MA, USA; #11181) was used. Transfections were performed using lipofectamine RNAiMax transfection reagent (13778-150, Invitrogen, Carlsbad, CA, USA) according to the manufacturer’s instructions. Briefly, the cells were seeded to 70% confluence in 10 cm dishes before transfections. The siRNAs were diluted to 10 nM of the final concentration in Opti-MEM and incubated with lipofectamine RNAiMax at a 1:1 ratio for 15 min. For transient expression, 5 μg of the plasmid was used. The siRNA-lipofectamine complex was then transferred to plated cells. Knockdown and induction of protein expression were verified by western blot analysis using specific antibodies.

### 4.6. Cell Cycle Analysis

For cell cycle analysis, cells were collected 24 h post-irradiation and fixed with pre-chilled 70% ethanol. The cells were incubated with 1 mg/mL RNase and 50 μg/mL propidium iodide (PI) in the dark for 30 min at 37 °C and subjected to flow cytometry using a BD FACSVerse flow cytometer (BD Biosciences, Franklin Lakes, NJ, USA). The cell cycle distributions were acquired and analyzed using BD FACSuite software.

### 4.7. Apoptosis Analysis

For the apoptosis assay, cells were collected 72 h post-irradiation and stained with annexin V-FITC (BD Pharmingen, San Diego, CA, USA) and 2 μg/mL PI in annexin V binding buffer (10 mM HEPES, pH 7.4, 140 mM NaCl, and 2.5 mM CaCl_2_) for 15 min at 37 °C in the dark. The apoptotic cell populations were analyzed using a BD FACSVerse flow cytometer and BD FACSuite software.

### 4.8. Western Blot Analysis

Western blot analyses were performed as previously described [37]. Briefly, the cell lysates were prepared in a modified RIPA buffer: 20 mM Tris, pH 7.4, 100 mM NaCl, 0.1% SDS, 1% NP-40, 1 mM EDTA, 1% Triton X-100, 0.5% deoxycholate, phosphatase inhibitor cocktail (phosSTOP, Roche, Basel, Switzerland; 04-906-837-001), and protease inhibitor cocktail (cOmplete tablets, Roche, 04-693-116-001). The cell lysates were centrifuged at 13,000 rpm for 30 min. The protein concentration in the supernatant was determined using a Bio-Rad protein assay reagent (Bio-Rad, Richmond, CA, USA) according to the manufacturer’s instructions. Equal amounts of proteins were separated by SDS-PAGE and transferred to nitrocellulose membranes (Bio-Rad). The blots were blocked overnight with 10% skim milk in TPBS at 4 °C and probed overnight with the primary antibodies listed above. After secondary antibody incubation, specific bands were detected using Amersham ECL western blotting detection reagents (GE Healthcare, Piscataway, NJ, USA).

### 4.9. Immunofluorescence Analysis

Fluorescence images were acquired and analyzed as previously described [35]. Briefly, 2 × 10^4^ cells were transfected with either control siRNA or *CCND1* siRNA and were seeded onto a coverglass (Marinfild Inc., Rochester, NY, USA) the next day. The cells were exposed to 4 Gy of X-rays or proton irradiation, followed by fixing with 4% formaldehyde for 4 or 24 h. After permeabilization and blocking processes, cells were incubated with RAD51 antibody (1:250) for 2 h, followed by incubating with Alexa Fluor 488-conjugated secondary antibody (Life Technologies, Paisley, UK) and 4′,6-diamidino-2-phenylindole (DAPI) for 30 min at room temperature. Fluorescence images were acquired using a fluorescence microscope (Zeiss Observer D1, Carl Zeiss Co., Ltd., Jena, Germany).

### 4.10. Statistics

Data are shown as the mean ± standard error of the mean (SEM) from at least two independent experiments. Statistical analyses were performed using GraphPad Prism 7.02. The statistical significance between experimental groups was calculated using a student *t*-test or a two-way ANOVA with Bonferroni correction. The statistical significance of the RBE was calculated using a one-sample *t*-test and comparing to the traditional generic RBE of 1.1. Values of *p* < 0.05 were considered statistically significant.

## 5. Conclusions

In the current study, we aimed to identify biomarkers that are associated with successful management of TNBC using proton beam therapy. For this, we screened for radiosensitivity and determined the RBE using clinical radiation beams (230 MeV protons and 6 MV X-rays). We identified cyclin D1 as a potential genetic factor that contributes to proton sensitivity in TNBC. Our findings may open up the opportunity for genome-based precision proton therapy. Although clinical validation of cyclin D1 for predicting proton therapy responses is warranted, our results show promise for a move toward individually-tailored proton beam therapy that considers both biological and physical factors.

## Supplementary Materials

Supplementary materials can be found at https://www.mdpi.com/1422-0067/20/19/4943/s1.

## Abbreviations

BC	breast cancer
CDK	cyclin-dependent kinase
CDKN2A	cyclin-dependent kinase inhibitor 2A
DSB	double-strand break
ER	estrogen receptor
FITC	fluorescein isothiocyanate
HER2	human epidermal growth factor receptor 2
NSCLC	non-small cell lung cancer
PARP	poly (ADP-ribose) polymerase
PR	progesterone receptor
RB	retinoblastoma susceptibility gene
RBE	relative biological effectiveness
RIPA	radio-immunoprecipitation assay
SDS-PAGE	sodium dodecyl sulfate-polyacrylamide gel electrophoresis
SF	survival fraction
siRNA	small interfering ribonucleic acid
SOBP	spread-out Bragg peak
TNBC	triple-negative breast cancer
