# Supplementary material for: Cyclin D1 is Associated with Radiosensitivity of Triple-Negative Breast Cancer Cells to Proton Beam Irradiation
Source: Int J Mol Sci. 2019 Oct 7;20(19):4943. doi: 10.3390/ijms20194943 (PMC6801441; doi:10.3390/ijms20194943)
Supplement: Supplementary file 1 [file ijms-20-04943-s001.pdf]

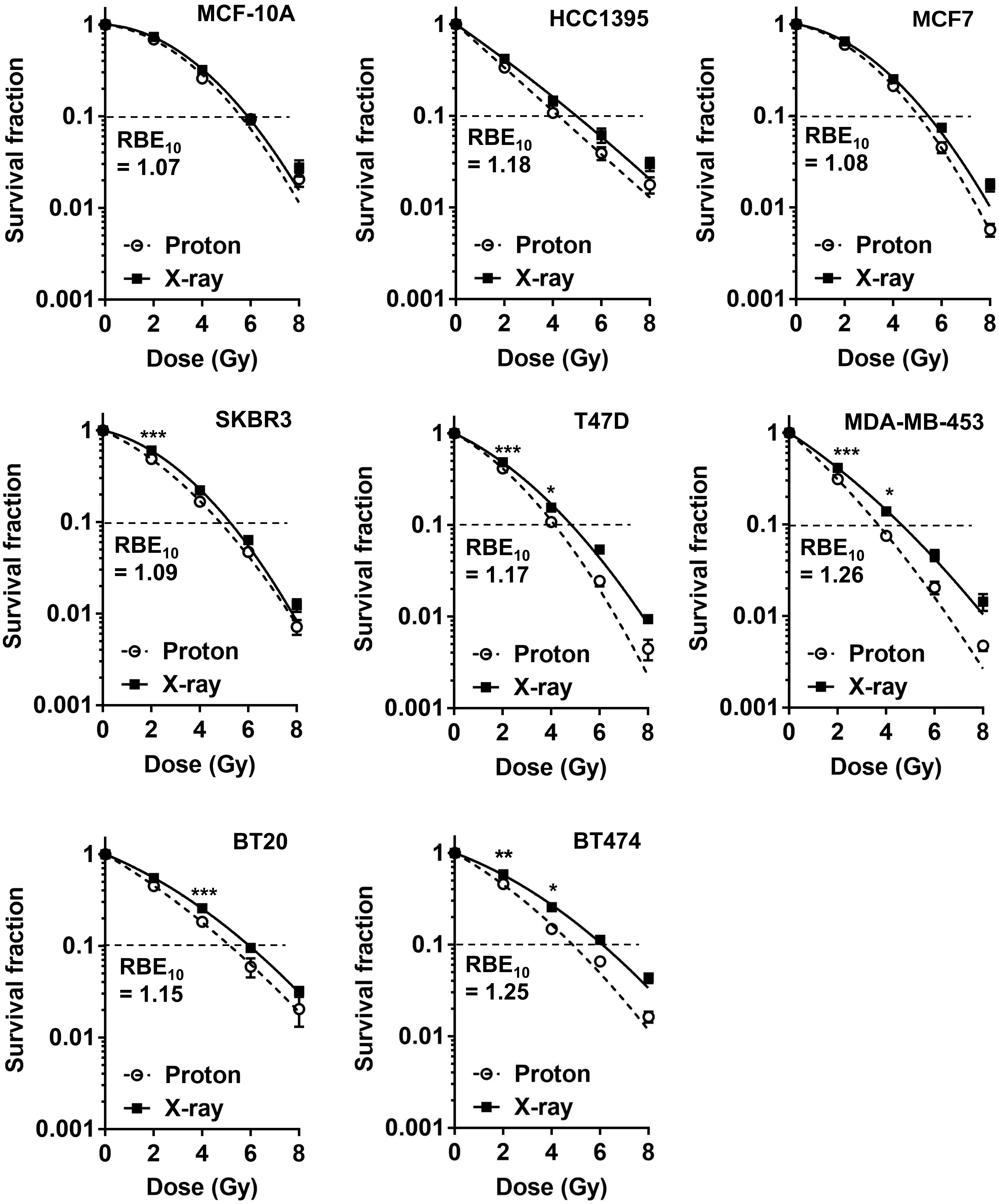

Figure S1. Clonogenic survival curves of eight human breast cancer cell lines against X-ray and proton irradiation. Data are presented as the mean ± SEM of three independent experiments. Curves were fitted with linear quadratic model. The differences are evaluated by a two-way ANOVA; *p < 0.05; *** p < 0.001. Dashed lines indicate SF = 0.1.

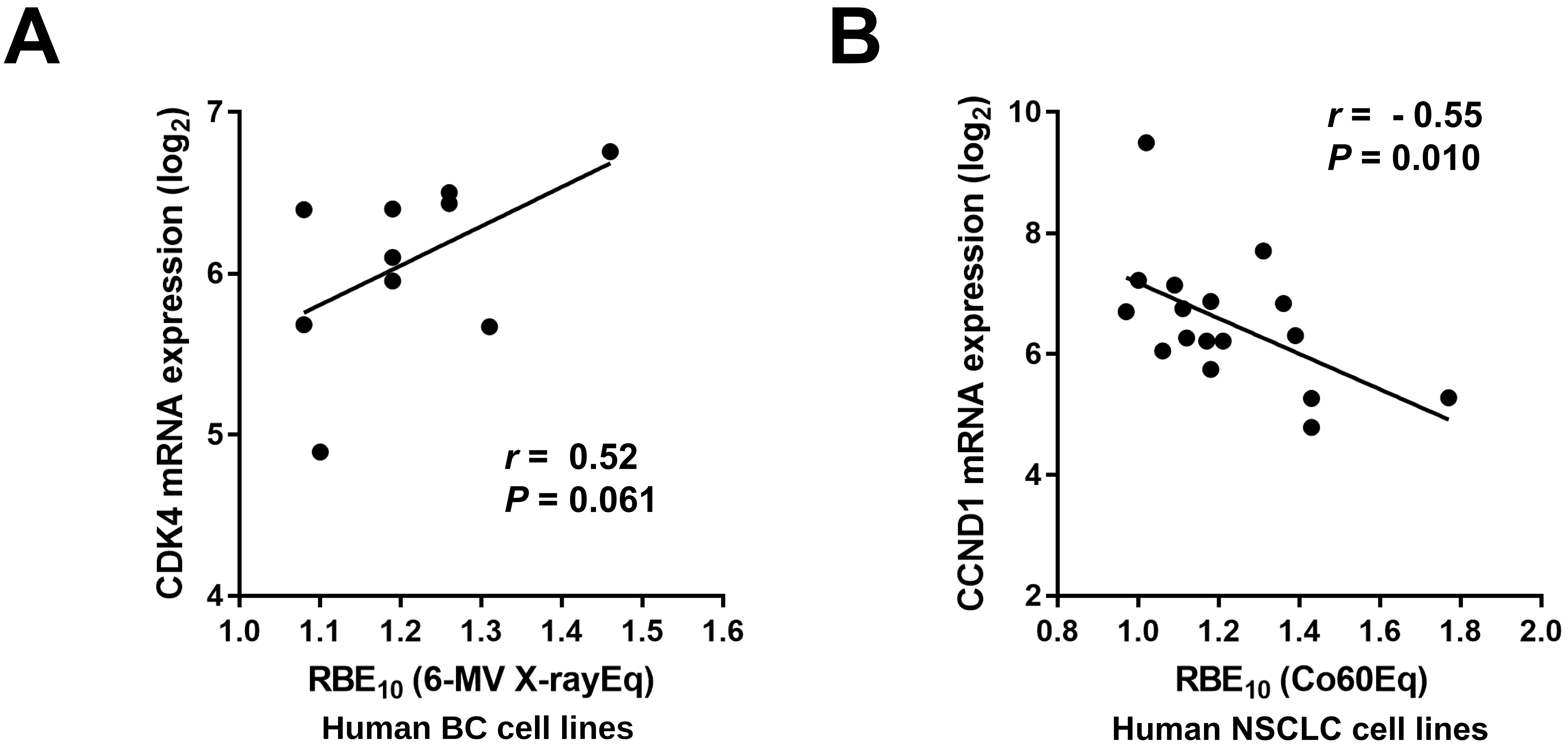

Figure S2. Scatter plot for correlation between RBE10 values and CDK4 mRNA expression in human BC cell lines (**A**) and cyclin D1 mRNA expression in human NSCLC cell lines (**B**). Log2 transformed RMA values were obtained from CCLE database. Pearson correlation coefficient (r) between gene expression and proton RBE10 across all samples were presented.
